# Enhancing Sensitivity in SARS-CoV-2 Rapid Antigen Testing through Integration of a Water-Soluble Polymer Wall

**DOI:** 10.3390/bios14060305

**Published:** 2024-06-12

**Authors:** Xiuzhen Wang, Yu Wang, Huiyang Jie, Sidi Liu, Chenguang Shen, Qian Liu

**Affiliations:** 1 School of Public Health, Southern Medical University, No. 1023, South Shatai Road, Baiyun District, Guangzhou 510515, China; wang_xiuzhen@gzlab.ac.cn; 2 Department of Detection and Diagnosis Technology Research, Guangzhou National Laboratory, Guangzhou 510000, China; wang_yu@gzlab.ac.cn (Y.W.); jie_huiyang@gzlab.ac.cn (H.J.); liusidi1990@outlook.com (S.L.)

**Keywords:** capillary time, lateral flow immunoassay, SARS-CoV-2, signal enhancement, water-soluble polymer wall

## Abstract

Lateral flow immunoassays (LFIAs) are recognized for their practicality in homecare and point-of-care testing, owing to their simplicity, cost-efficiency, and rapid visual readouts. Despite these advantages, LFIAs typically fall short in sensitivity, particularly in detecting viruses such as SARS-CoV-2, thus limiting their broader application. In response to this challenge, we have innovated an approach to substantially enhance LFIA sensitivity. This involves the integration of a water-soluble dextran–methacrylate polymer wall with a 15% grafting degree positioned between the test and control lines on the LFIA strip. This novel modification significantly improved the sensitivity of the assay, achieving detection limits as low as 50 pg mL^−1^ and enhancing the sensitivity by 5–20-fold relative to existing LFIA kits available on the market. Furthermore, our developed LFIA kit (WSPW-LFIA) demonstrated exceptional specificity for SARS-CoV-2. Coupled with a straightforward fabrication process and robust stability, the WSPW-LFIA represents a promising advancement for real-time in vitro diagnosis across a spectrum of diseases.

## 1. Introduction

Lateral flow immunoassays (LFIAs) play a pivotal role in paper-based biosensor technology, utilizing detection and capture antibodies strategically dispersed across test strips [1,2,3]. In these assays, analytes conjugated with detection antibodies are transported via capillary action across a nitrocellulose membrane to capture antibodies located at the Test line [2,4]. This technology has gained widespread recognition in point-of-care testing for diverse disease diagnoses, attributable to its simplicity, cost-effectiveness, and rapid visual readout [1,2,4,5,6,7]. Notably, during the COVID-19 pandemic, various biosensors for detecting SARS-CoV-2 antigens of spike proteins and nucleoproteins were reported and achieved high sensitivity levels of 2.1 pg mL^−1^ to 1000 pg mL^−1^, like electrical [8,9], optical [10], luminescence [11], electrochemical [12,13,14] and fluorescence [15,16] biosensors. However, these methods are limited for homecare and point-of-care applications due to the disadvantages that they need to be performed in a controlled laboratory, there are multiple steps in the detecting procedure, they require equipment and trained technicians, and they are time-consuming. Conversely, LFIA antigen test kits based on the colorimetric approach possess the advantages of simplicity, a low cost, and results that are readable by the naked eye, and have become indispensable tools in both homecare and point-of-care settings [5,6,7]. Despite these benefits, LFIAs exhibit a lower sensitivity relative to nucleic-acid-based methods [17,18]. This reduced sensitivity can lead to false negatives, potentially delaying the control of virus transmission [19,20].

Consequently, enhancing the sensitivity of LFIA-based infectious disease test kits is paramount [5,17,18]. Strategies to improve LFIA sensitivity typically focus on manipulating the transport dynamics of the immune reaction under capillary force [2,4,21]. Several methods aimed at controlling fluidic dynamics have been investigated. For instance, modifying the LFIA structure by adding a sponge shunt can slow down the flow of fluids [22], or changing the sample and conjugate pads may direct fluid movement more effectively [23]. Other explored strategies include using sugar or salt barriers to adjust fluid timing and flow [24,25]. Using a dissolvable wax structure has also been studied to delay fluid movement [26,27]. However, these improvements still come with the drawbacks of the complexity of the manufacturing process, leading to high costs; the fact that antigen–antibody binding interactions are affected by alterations in the ionic strength after sugar or salt dissolution; and sensitivity levels as low as 14,470 pg mL^−1^ [22,23,24,25,26,27].

In this study, we introduce a novel technique to significantly enhance the sensitivity of LFIAs by incorporating a water-soluble polymer wall (WSPW) into a nitrocellulose membrane. This innovative modification is designed to extend the duration of capillary action, thus improving the efficiency of the binding interactions within the immune response between antibodies and antigens. Our newly developed SARS-CoV-2 rapid antigen test kit, based on this WSPW-LFIA approach, has shown promising results in practical applications, meeting the stringent requirements for in vitro diagnosis in terms of sensitivity, specificity, and stability.

## 2. Materials and Methods

### 2.1. Materials and Equipments

The key reagents and materials used in our study were sourced from reputable suppliers to ensure high-quality and reliable results. The nucleoproteins of SARS-CoV-2, anti-COVID-19 detection antibodies, anti-COVID-19 capture antibodies, and goat-anti-mouse antibodies were procured from Fapon (Dongguan, China). Bovine serum albumin (BSA) was obtained from GBCBIO (Guangzhou, China). Polyethylene glycol 4000, polyethylene glycol 2000, and Dextran6000 were acquired from Macklin (Shanghai, China). Polyvinyl alcohol and polyethylene glycol diacrylate 6000 were supplied by Sigma-Aldrich (St. Louis, MO, USA). For the preparation of nanoparticles, key chemicals such as tetrachloroauric acid and trisodium citrate were acquired from Macklin (Shanghai, China). The nitrocellulose membrane essential for our lateral flow assay was purchased from Tian Ren (Suzhou, China). The fiberglass membrane and absorption pad were sourced from Shanghai Kinbio Tech (Shanghai, China).

Inactivated viral cultures of five respiratory viruses (influenza A H1N1, influenza A H3N2, influenza B Yamagata, parainfluenza, and respiratory syncytial virus) were purchased from Bioantibody Biotech (Nanjing, China). SARS-CoV-2 rapid antigen test kits were acquired from Wondfo Biotech (Guangzhou, China), Orient Gene Biotech (Huzhou, China), and ACON Biotech (Hangzhou, China). Seven strains of SARS-CoV-2 (wild type, alpha, beta, delta, gamma, omicron-BA.1, and omicron-BA.2) were donated by the Guangdong Provincial Center for Disease Control and Prevention (Guangdong CDC).

The HM 3030 dispenser machine and HGS220 cutter machine were from Hangzhou Fenghang Tech (Hangzhou, China); the Pharos G1 scanning electron microscope (SEM) was from Phenomchina Cnpowder (Shanghai, China), the Nano-500 UV/vis spectrophotometer was from Allsheng (Hangzhou, China); and the Tecnai G2 Spirit transmission electron microscope (TEM) was from Thermo Fisher Scientific (Carlsbad, CA, USA). The iBright™ FL1500 Imaging System was from Thermo Fisher Scientific (Carlsbad, CA, USA).

### 2.2. Preparation of AuNPs and AuNP–Antibody Conjugates

The method for synthesizing the gold nanoparticles (AuNPs) followed a previous study [28,29]. The synthesis of AuNPs was conducted via the reduction of tetrachloroauric acid using trisodium citrate. In this process, 4 mL of HAuCl_4_ solution (1.171 wt%) was added to a flask containing 100 mL of distilled water under constant stirring. Upon boiling, 1.5 mL of trisodium citrate solution (4.08 wt%) was introduced. The solution was removed from the heat once it turned deep red and was allowed to cool. The AuNP solution was then adjusted to pH 8.0 using potassium carbonate (0.2 M) and combined with 20 µg of anti-COVID-19 detection antibodies, followed by incubation at 25 °C for 10 min. BSA (20 µL, 10 wt%) was subsequently added, and the mixture was incubated for another 5 min at 25 °C for blocking. After centrifugation at 5000 rcf for 20 min at 25 °C, excess antibodies and BSA were removed. The AuNP–antibody conjugates were then resuspended and stored in a 50 µL conjugate solution containing NaCl (1 wt%), Tween 20 (0.5 wt%), BSA (1 wt%), sucrose (10 wt%), and TRIS (10 mM, pH 8.0).

The analytical characterization of our AuNPs and AuNP–antibody conjugates was meticulously conducted. The morphology and distribution of the AuNPs were examined through a TEM [27,30]. The AuNPs showed a spherical shape and a uniform dispersibility, with an average diameter of 27 nm in Figure S1A. Additionally, the AuNP–antibody conjugates were characterized by a redshift in their UV absorption. As shown in Figure S1B, compared with AuNPs, the maximum absorption of AuNP–antibody conjugates displayed a redshift of 6 nm from 529 nm to 535 nm, which indicates the successful conjugation of antibodies [27,31].

### 2.3. Fabrication of LFIA Test Strips

The LFIA test strip in our study contained three key components: the sample pad, the conjugate pad, and the nitrocellulose membrane. The sample pad underwent a treatment process with a solution containing NaCl (1 wt%), Tween 20 (0.5 wt%), BSA (2 wt%), and TRIS (10 mM, pH 8.0), followed by a drying phase in an oven at 37 °C for 12 h. The conjugate pad was prepared by dispensing the conjugate solution onto a fiberglass membrane, which was then dried in an oven at 37 °C for 2 h. For the nitrocellulose membrane, it was treated with Test line solution containing anti-COVID-19 capture antibodies (2 mg mL^−1^), sucrose (2 wt%), and PBS (10 mM, pH 7.2), and Control line solution containing goat-anti-mouse antibodies (0.8 mg mL^−1^), sucrose (2 wt%), and PBS (10 mM, pH 7.2). These solutions were precisely dispensed onto the nitrocellulose membrane, maintaining a distance of 5 mm between the Test and Control lines. The membrane was then dried at 37 °C for 4 h. Upon preparation of these materials, they were assembled on a card and sectioned into individual test strips, each measuring 4 mm in length.

### 2.4. Statistics and Analysis of Results

Images of the test strip were captured using an iBright™ FL1500 Imaging System and subsequently analyzed using ImageJ software (version 1.4.3.67) (NIH, Bethesda, MD, USA). The analysis followed the guidelines outlined in CLSI EP17, a document providing standard procedures for evaluating the detection capabilities of clinical laboratory measurements. The data are presented as means ± standard deviation (SD), and the significance of differences between groups was determined using one-way ANOVA with Tukey’s post hoc test. A *p*-value of less than 0.05 was considered statistically significant.

## 3. Results and Discussion

### 3.1. Design of the WSPW-LFIA Assay

**Figure 1:**
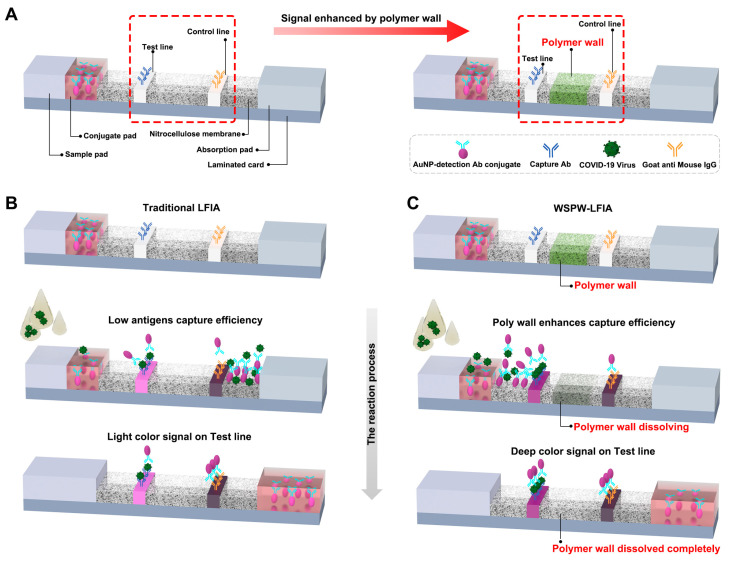
Illustration of the LFIA strip’s operational mechanism. (**A**) Structure and key components of the WSPW-LFIA strip, highlighting the placement of the polymer wall between the Test line and Control line. (**B**) Traditional LFIA antigen detection process, depicting the low capture efficiency and the resulting low sensitivity. (**C**) Enhanced antigen detection in WSPW-LFIA, showcasing how the integrated polymer wall improves the capture efficiency by retaining antigens and antibodies at the test line.

The LFIA comprises five critical components: a pretreatment sample pad, a conjugate pad, a nitrocellulose membrane with designated Test and Control lines, an absorption pad, and a laminated card (illustrated in Figure 1A). In the detection process, the sample lysis buffer containing antigens travels to the Test line through capillary action after being added to the sample pad. A visible color will be observed by the naked eye, accompanied by the binding between AuNP–antibody–antigen conjugates and the capture of antibodies on the Test line. In a traditional LFIA setup (Figure 1B), without the integration of a polymer wall, a low antigen-capture efficiency can result in a lower sensitivity. Conversely, in the WSPW-LFIA configuration (Figure 1C), a polymer wall is integrated between the Test line and the Control line. During the detection process, the sample flow will be retained around the Test line until the polymer wall is completely dissolved. This results in signal enhancement due to improving the efficiency of the immunoreaction reaction by prolonging the immunoreaction time.

### 3.2. Construction of WSPW-LFIA

Our preliminary evaluation focused on selecting suitable polymers for the WSPW-LFIA. We considered seven types of polymers, including hydrolyzed polyvinyl alcohol (hydrolyzed PVA), polyvinyl alcohol (PVA), polyethylene glycol diacrylate 6000 (PEGDA6000), polyethylene glycol 4000 (PEG4000), polyethylene glycol 2000 (PEG2000), dextran–methacrylate with a 15% grafting degree (DexMA15), and Dextran6000 (Dex6000). All these materials possess the essential properties of water solubility, biocompatibility, and protein resistance. Previous research has demonstrated the effectiveness of polyvinyl alcohol, polyethylene glycol, and dextran in modifying mesoporous materials for biological field applications [32,33,34,35,36].

**Figure 2:**
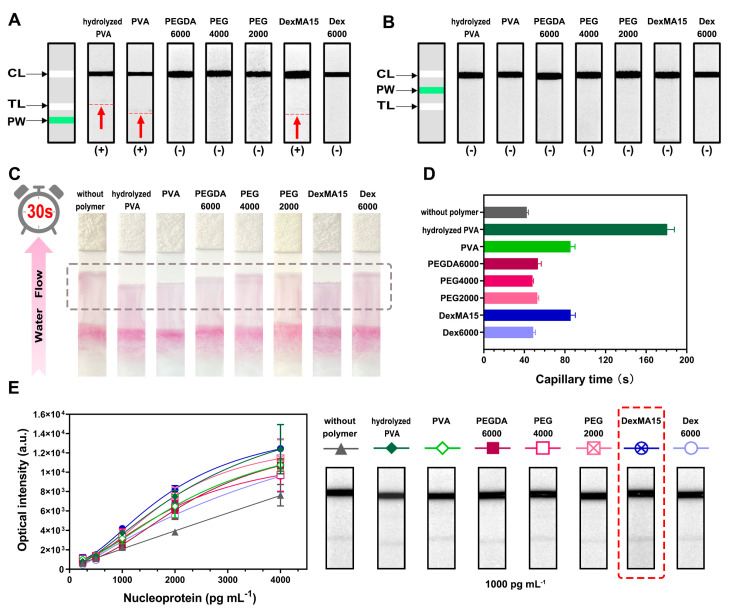
Comparison of LFIA strips with varied polymer wall integration. (**A**) Strips with a polymer wall positioned ahead of the Test line for SARS-CoV-2 nucleoprotein detection (0 pg mL^−1^). (**B**) Strips with a polymer wall situated between the Test and Control lines for SARS-CoV-2 nucleoprotein detection (0 pg mL^−1^). (**C**) Displacement distances of strips featuring different polymer walls, measured 30 s post sample introduction. (**D**) Capillary times for strips with diverse polymer walls, timed from sample introduction to the conjugate pad until reaching the control line. (**E**) Detection of SARS-CoV-2 nucleoprotein. (Standard deviations represented over three measurements of all the detections).

In this study, we explored the effect of various WSPWs on the performance of the LFIA for SARS-CoV-2 detection. The materials selected for the polymer walls were strategically positioned either at the front or behind the Test line on nitrocellulose membranes. As illustrated in Figure 2A,B, placing the polymer walls, specifically those made of hydrolyzed PVA, PVA, and DexMA15, in front of the Test line resulted in false positive signals. In contrast, positioning these polymer walls behind the Test line eliminated such false positives, highlighting the importance of their strategic placement.

Further evaluation was conducted to assess the impact of these polymer walls on the capillary time of the sample on nitrocellulose membranes. Figure 2C demonstrates that the sample flow was quicker on strips without a polymer wall, measured within 30 s, compared to those with polymer walls. Notably, the strips with hydrolyzed PVA, PVA, and DexMA15 polymer walls exhibited the slowest flow speed, indicated by the shortest displacement distance. Additionally, the presence of polymer walls influenced the capillary time when the sample flow interacted with the capture antibodies on the Test line. As Figure 2D shows, the capillary time significantly increased from 42 s (without polymer walls) to 86 s (with PVA and DexMA15 polymer walls), and even to 181 s (with a hydrolyzed PVA wall). This finding indicates that the capillary time can be effectively modulated by the incorporation of a polymer wall between the Test and Control lines.

The efficacy of LFIAs for SARS-CoV-2 detection using different WSPWs was further explored. As depicted in Figure 2E, LFIAs both without and with various polymer walls, including hydrolyzed PVA, PVA, PEGDA6000, PEG4000, PEG2000, DexMA15, and Dex6000, were separately constructed. In our study, we observed a marked enhancement in the detection capabilities of LFIAs for SARS-CoV-2 when incorporating polymer walls. With increasing concentrations of SARS-CoV-2 nucleoproteins, ranging from 250 pg mL^−1^ to 4000 pg mL^−1^, the LFIAs with polymer walls exhibited significantly stronger positive signals compared to those without polymer walls. Notably, the LFIA incorporating a DexMA15 polymer wall showed a superior signal intensity relative to LFIAs with other polymer types. Despite hydrolyzed PVA and PVA providing comparable or longer capillary times, DexMA15’s unique attributes—a higher polymer molecule concentration, a longer polymer chain length, and methacrylate grafting—enhance its steric repulsion from proteins. This repulsion likely causes antigen–antibody complexes to accumulate near the Test line, enhancing the detection sensitivity. This mechanism and the effect of steric repulsion between polymers and proteins have been substantiated by previous studies [37,38].

Linear correlation analyses comparing LFIAs with and without polymer walls are shown in Table S1 and further support the enhanced detection of SARS-CoV-2 through the use of the DexMA15 polymer wall. The use of this water-soluble polymer to create a wall between the Test and Control lines effectively prolongs the reaction time between antigens and antibodies, thereby boosting the assay’s sensitivity.

### 3.3. Optimization of WSPW-LFIA with DexMA15

**Figure 3:**
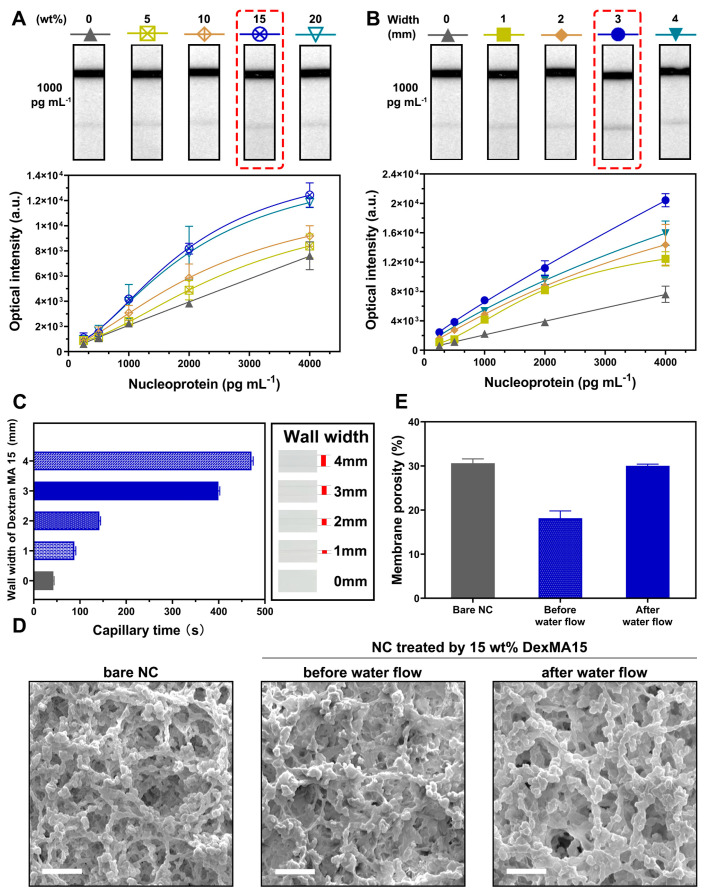
Sensitivity analysis of the LFIA with DexMA15. (**A**) LFIA with varying concentrations of DexMA15 for detecting SARS-CoV-2 nucleoproteins. (**B**) LFIA with different widths of DexMA15 for detecting SARS-CoV-2 nucleoproteins. (**C**) Capillary times for LFIAs with varying widths of DexMA15. (**D**) SEM images comparing nitrocellulose membranes with and without DexMA15 treatment. Scale bars represent 10 μm. (**E**) Porosity comparison of nitrocellulose membranes with and without DexMA15 treatment under different sample flow conditions. (Standard deviations derived from three measurements of all the detections).

To optimize the performance of the WSPW-LFIA, DexMA15 was identified as the most suitable material, and its parameters were meticulously fine-tuned. We prepared antigen test strips with varying concentrations of DexMA15 (0–20 wt%) and a consistent width of 1 mm. As illustrated in Figure 3A, the sensitivity of the signal for detecting SARS-CoV-2 nucleoprotein improved as the concentration of DexMA15 was increased from 250 pg mL^−1^ to 4000 pg mL^−1^. Compared to the test strips containing 0, 5, and 20 wt% DexMA15, the test strip treated with 15 wt% DexMA15 demonstrated the highest signal intensity and the most distinct visual color change, making it easily identifiable by the naked eye. Additionally, test strips integrated with varying widths (from 0 to 4 mm) of 15 wt% DexMA15 were fabricated to assess their detection performance. In our experimental setup, as depicted in Figure 3B, we observed a direct correlation between the width of the DexMA15 and the signal intensity in the detection of SARS-CoV-2 nucleoprotein across a concentration range of 250 pg mL^−1^ to 4000 pg mL^−1^. The signal intensity increased progressively with the widening of the DexMA15 from 0 to 4 mm. Notably, at a width of 3 mm, the color intensity at the Test line was markedly deeper and the signal strength was significantly higher compared to other tested conditions. The capillary time for the test strips containing DexMA15 of various widths was also meticulously measured. As shown in Figure 3C, the strips with 3 mm and 4 mm polymer walls exhibited substantially longer capillary times, reaching 400 s and 471 s, respectively, far exceeding those of narrower walls. While a wider DexMA15 wall provides extended capillary times for the antigen–antibody immune reaction, it was observed that the strip with a 4 mm wall, despite having the longest capillary time, exhibited a reduced sensitivity due to the reversibility of the immune reaction. Consequently, the test strip with 3 mm wide DexMA15 emerged as the most effective, achieving the highest signal intensity.

To further understand the underlying mechanism, we investigated the porosity changes of the nitrocellulose membrane before and after the incorporation of DexMA15 using an SEM, as illustrated in Figure 3D. The SEM analysis revealed that most pores in the nitrocellulose membrane were effectively filled upon the addition of DexMA15. When the sample flow was introduced, the membrane’s porosity was restored due to the water-soluble nature of DexMA15. A quantitative analysis, shown in Figure 3E, indicated that the membrane porosity decreased from 30.62% to 18.16% after being treated with 15 wt% DexMA15 and subsequently recovered to 30.02% following the introduction of the sample flow. These findings demonstrate that the capillary time can be precisely controlled by filling an optimal amount of DexMA15 into the pores of the nitrocellulose membrane, thereby enhancing the performance of the LFIA for SARS-CoV-2 antigen detection.

### 3.4. Detection of WSPW-LFIA with DexMA15

**Figure 4:**
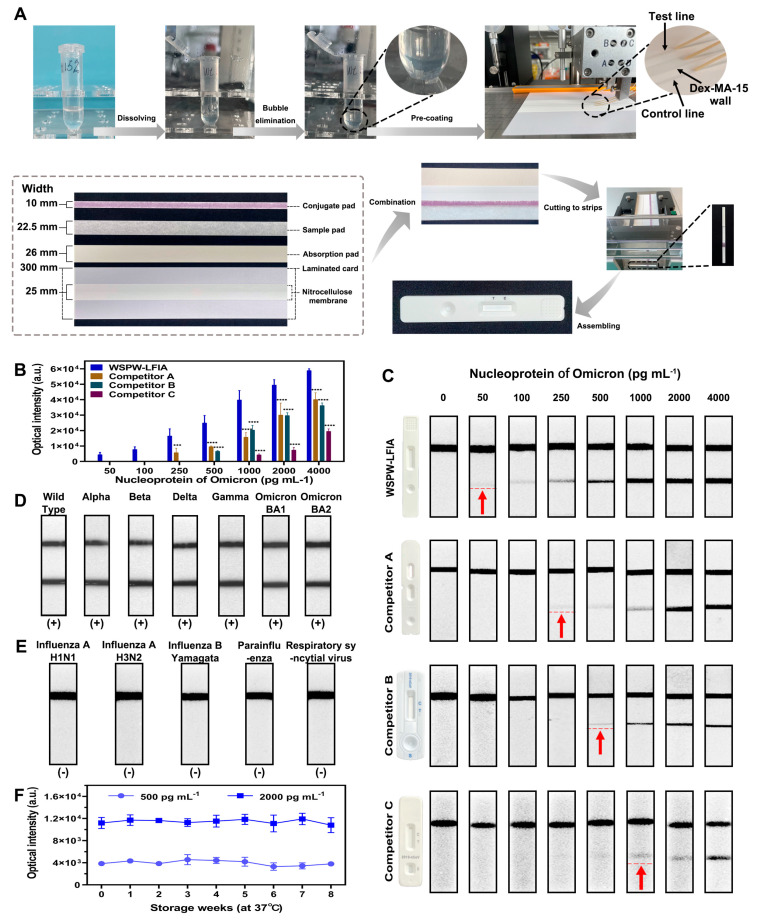
Evaluation of the WSPW-LFIA-based SARS-CoV-2 rapid antigen test kit. (**A**) Fabrication process of the test kit. (**B**) Comparative detection of SARS-CoV-2 nucleoproteins using the WSPW-LFIA and three commercial products. The results were analyzed using ImageJ software. Standard deviations and statistical significance (*** *p* < 0.001, **** *p* < 0.0001) were calculated over three measurements, using one-way ANOVA with Tukey’s post hoc test. (**C**) Images of test strips post detection of SARS-CoV-2 nucleoproteins. (The red arrow indicates the detection limit of the test kit). (**D**) Detection of various SARS-CoV-2 strains (wild type, alpha, beta, delta, gamma, omicron-BA.1, and omicron-BA.2) at a concentration of 10^2^ TCID50. (**E**) Detection of five respiratory viruses (influenza A H1N1, influenza A H3N2, influenza B Yamagata, parainfluenza, and respiratory syncytial virus) at a concentration of 10^4^ TCID50. (**F**) Stability assessment of test strips stored at 37 °C over 8 weeks, with each positive concentration tested at least three times. Statistics are calculated based on CV, where CV = (standard deviation/mean) × 100. Standard deviations from three measurements are indicated.

To validate the performance of the WSPW-LFIA integrated with DexMA15, we developed a test kit and assessed its performance. The mass production process, illustrated in Figure 4A, involved the application of DexMA15 and the capture of antibodies onto a nitrocellulose membrane to form the water-soluble wall and the Test line, respectively. The assembly of the test strip was completed by cutting and laminating the conjugated pad, sample pad, absorption pad, laminated card, and the prepared nitrocellulose membrane.

The key parameters for in vitro diagnostics—sensitivity, specificity, and stability—were evaluated, with the results presented in Figure 4. The sensitivity of the WSPW-LFIA with DexMA15 was benchmarked against three commercial SARS-CoV-2 antigen test kits. These tests were employed to detect varying concentrations of Omicron nucleoproteins, ranging from 50 pg mL^−1^ to 4000 pg mL^−1^. Figure 4B,C depict that the WSPW-LFIA with DexMA15 exhibited a significantly higher positive signal intensity compared to the three competitor kits. Furthermore, it demonstrated excellent linearity in detecting low antigen concentrations. Remarkably, the WSPW-LFIA with DexMA15 achieved a detectable cut-off limit visible to the naked eye at 50 pg mL^−1^. This sensitivity is 5–20 times greater than that of the competing products, with competitor A (Wondfo) at 250 pg mL^−1^, competitor B (Orient Gene) at 500 pg mL^−1^, and competitor C (ACON) at 1000 pg mL^−1^. These findings underscore the superior performance of the WSPW-LFIA with a DexMA15 wall in terms of sensitivity.

To verify the specificity of the WSPW-LFIA in detecting real samples, cultures of inactivated viruses were utilized to simulate clinical samples. As shown in Figure 4D,E, seven inactivated SARS-CoV-2 variants (wild type, alpha, beta, delta, gamma, omicron-BA.1, and omicron-BA.2) were treated as positive samples, and five other respiratory viruses (influenza A H1N1, influenza A H3N2, influenza B Yamagata, parainfluenza II, and respiratory syncytial virus) were treated as negative samples. All of the cultures of inactivated viruses are from Guangdong CDC and Bioantibody Biotech. The results show that all the SARS-CoV-2 variants were positive, and the five other respiratory viruses were negative, which indicates the good specificity of the WSPW-LFIA. The results of seven SARS-CoV-2 strains at concentrations ranging from 0.1 to 10^2^ TCID_50_ are presented in Supplementary Figure S2.

Furthermore, the stability of the test kit was assessed over an eight-week period at a storage temperature of 37 °C, using samples at two positive levels (500 and 2000 pg mL^−1^). The results, depicted in Figure 4F and Supplementary Figure S3, were analyzed using the coefficient of variation (CV), a statistical measure that describes the relative variability of a dataset in relation to its mean. The analysis showed that 100% of the test strips retained their activity, with a CV of less than 20% at 500 pg mL^−1^ and less than 10% at 2000 pg mL^−1^. These results underscore that the WSPW-LFIA with a DexMA15 wall possesses broad potential for commercial application, characterized by its ease of fabrication and exceptional sensitivity, specificity, and stability. This positions the WSPW-LFIA-based SARS-CoV-2 rapid antigen test kit as a highly effective tool for the detection of SARS-CoV-2 in various clinical and point-of-care settings.

The properties of different sensors for detecting SARS-CoV-2 reported in the literature have been summarized in Table S2; the sensitivity of the WSPW-LFIA in detecting nucleoproteins of SARS-CoV-2 is higher than other sensors, like electrical, electrochemical and fluorescence sensors. In addition, the WSPW-LFIA presents various advantages such as a simple operation, without additional reading equipment, and rapidity.

## 4. Conclusions

In this study, we successfully demonstrated a strategy to significantly enhance LFIAs’ signal sensitivity for SARS-CoV-2 detection (to as low as 50 pg mL^−1^) by integrating a wall of dextran–methacrylate with a 15% grafting degree (DexMA15) onto a nitrocellulose membrane. This innovation enhanced the capture efficiency through manipulating the capillary time to up to 400 s, resulting in a 5–20-fold increase in sensitivity compared to three commercially available test strips. The utility of our approach has been thoroughly validated, fulfilling the stringent criteria of in vitro diagnostics in terms of sensitivity, specificity, and stability. The fabrication process employed in our study maintains cost-effectiveness and simplicity, ensuring that the end product is not only affordable but also user-friendly. These characteristics render our enhanced-sensitivity solution particularly promising for the future development and refinement of diagnostic devices for various diseases. The ease of use and affordability of the LFIA, augmented by our novel polymer wall integration strategy, position it as an exemplary tool for homecare and point-of-care applications, particularly in the detection of infectious diseases and the monitoring of chronic conditions.

## Data Availability

Data is contained within the article and Supplementary Materials.

## Supplementary Materials

The following supporting information can be downloaded at: https://www.mdpi.com/article/10.3390/bios14060305/s1, Figure S1: Characterization of AuNPs and AuNP-antibody conjugates; Figure S2: Sensitivity analysis of seven SARS-CoV-2 strains; Figure S3: Stability assessment over 8 weeks with DexMA15. Table S1: Comparative results of LFIA for SARS-CoV-2 nucleoprotein detection without and with different polymer walls; Table S2: Summarization of biosensors for detecting SARS-CoV-2 antigens in literatures.
