# Supplementary material for: Enhancing Sensitivity in SARS-CoV-2 Rapid Antigen Testing through Integration of a Water-Soluble Polymer Wall
Source: Biosensors (Basel). 2024 Jun 12;14(6):305. doi: 10.3390/bios14060305 (PMC11201958; doi:10.3390/bios14060305)
Supplement: Supplementary file 1 [file biosensors-14-00305-s001.zip › biosensors-2997247-supplementary.pdf]

Supplementary Material

Enhancing Sensitivity in SARS-CoV-2 Rapid Antigen Testing through Integration of a Water-Soluble Polymer Wall

Xiuzhen Wang [1,2], *Yu Wang* [2], *Huiyang Jie* [2], *Sidi Liu* [2], *Chenguang Shen* [1,*] *and Qian Liu* [2,*]

1 School of Public Health, Southern Medical University, No.1023, South Shatai Road, Baiyun District, Guangzhou, Guangdong 510515, China

2 Department of Detection and Diagnosis Technology Research, Guangzhou National Laboratory, Guangzhou, Guangdong, 510000, P. R. China

* Qian Liu: liu_qian2@gzlab.ac.cn; Chenguang Shen a124965468@smu.edu.cn

Table of Contents

1. **Supporting Figures**

2. **Supporting Tables**

1.Supporting Figures

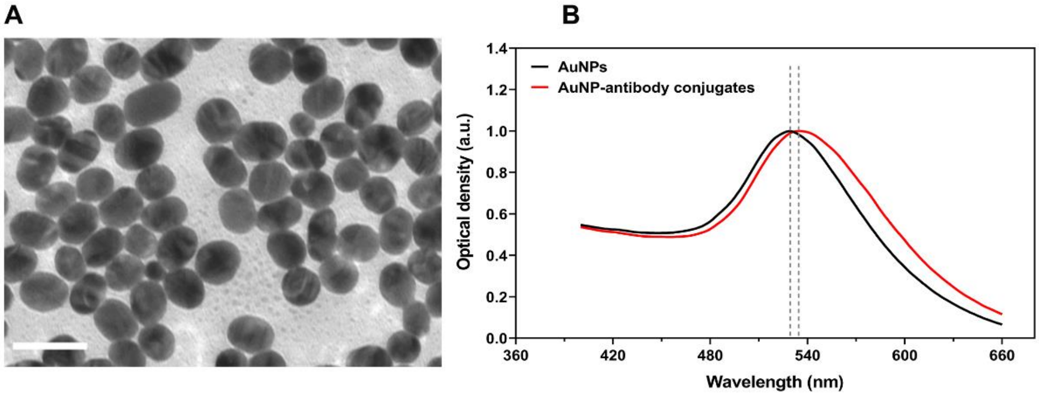

Figure S1. Characterization of AuNPs and AuNP-antibody conjugates. (**A**) TEM image of the AuNPs showed that the AuNPs have spherical shape and uniform dispersibility an average diameter of 27 nm. The image includes a 50 nm scale bar for reference. (**B**) The AuNP-antibody conjugates were characterized by the redshift of their UV absorption. Compared with AuNPs, the maximum absorption of AuNP-antibody conjugates displayed a redshift of 6 nm from 529 nm to 535 nm, which indicates the successful conjugation of antibodies.

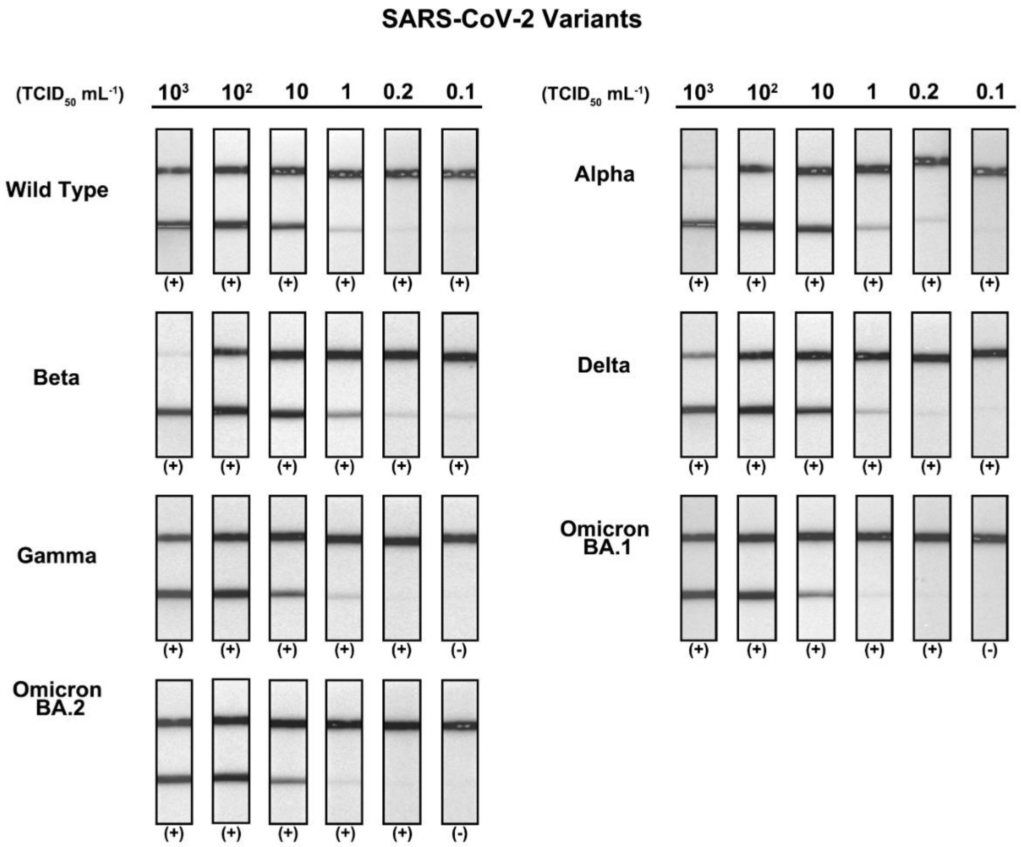

Figure S2. Sensitivity analysis of seven SARS-CoV-2 strains. Tests included wild-type, alpha, beta, delta, gamma, omicron-BA.1, and omicron-BA.2 variants, with serial dilutions ranging from 10^3 $TCID_{50}$ mL^{-1} to 0.1 $TCID_{50}$ mL^{-1}. Sensitivity levels for wild-type, alpha, beta, and delta strains reached as low as 0.1 $TCID_{50}$ mL^{-1}, while gamma, omicron-BA.1, and omicron-BA.2 showed a sensitivity of 0.2 $TCID_{50}$ mL^{-1}.

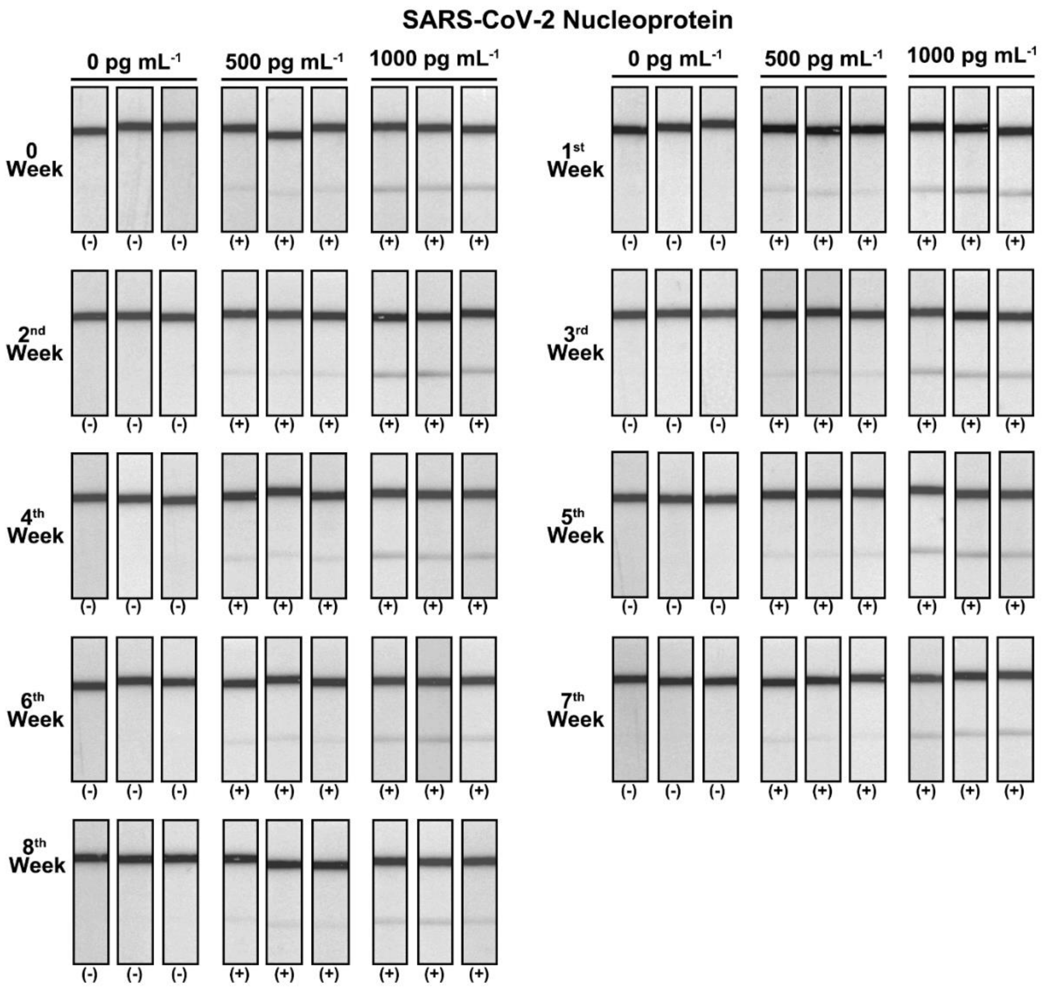

Figure S3. Stability assessment over 8 weeks with DexMA15. Test strips stored at 37°C were evaluated using three different concentrations of SARS-CoV-2 nucleoprotein (0 pg mL^{-1}, 500 pg mL^{-1}, and 2000 pg mL^{-1}) over a period of 0–8 weeks.

2.Supporting Tables

Table S1 Comparative results of LFIA for SARS-CoV-2 nucleoprotein detection without and with different polymer walls.

Detection of SARS-CoV-2 nucleoprotein								
Test Strip	Sample	250 pg mL^{-1}	500 pg mL^{-1}	1000 pg mL^{-1}	2000 pg mL^{-1}	4000 pg mL^{-1}	R^2	k
without polymer	1st	478	1538	2126	3668	8196	0.9700	1.845
	2nd	637	989	2253	3987	8306		
	3rd	710	876	2349	3812	6337		
hydrolyzed PVA	1st	686	1318	3974	6871	13995	0.9457	3.054
	2nd	982	1288	3345	8152	12273		
	3rd	679	1429	3895	7362	9895		
PVA	1st	1155	1264	3790	6107	10649	0.9670	2.660
	2nd	835	1050	3057	5507	11314		
	3rd	1058	1763	2687	7798	10263		
PEGDA6000	1st	881	1635	1976	6235	10795	0.9789	2.728
	2nd	816	904	2747	5100	10962		
	3rd	473	1033	2739	6969	10304		
PEG4000	1st	1401	1426	3206	6006	9066	0.9264	2.365
	2nd	1085	1277	2510	5996	8426		
	3rd	692	1402	4231	7007	11627		
PEG2000	1st	955	1587	3777	6671	12856	0.9315	2.900
	2nd	857	1075	3758	8468	9226		
	3rd	789	1464	2599	7404	12222		
DexMA15	1st	1540	1611	4383	8161	13408	0.9588	3.105
	2nd	927	1394	3872	8614	11467		
	3rd	806	1448	4278	7755	12439		
Dex6000	1st	361	935	2297	5062	7803	0.9450	2.438
	2nd	472	1229	3777	5651	9898		
	3rd	604	680	3216	5772	11107		

Table S2 Summarization of biosensors for detecting SARS-CoV-2 antigens in literatures.

Methods	Analytes	Limit of detection	Result readout	Time	Detection procedure	References
WSPW-LFIA	nucleoprotein	50 pg/ml	naked eye	15-30min	*	This work
Electrical	spike protein	10 pg/ml	eqiupment	20min	***	[8]
Electrical	nucleoprotein	136.25 pg/ml	eqiupment	30min	*	[9]
Optical	spike protein	> 100 pg/ml	eqiupment	20min	***	[10]
Luminescence	spike protein	2.1 pg/ml	eqiupment	40min	****	[11]
Electrochemical	nucleoprotein	0.1 ng/ml	eqiupment	60min	****	[12]
Electrochemical	spike protein	0.11 ng/ml	eqiupment	30min	**	[13]
Electrochemical	nucleoprotein	0.227 ng/ml	eqiupment	5-10min	***	[14]
Fluorescence	nucleoprotein	119 pg/ml	eqiupment	2.25h	****	[15]
Fluorescence	spike protein	1 ng/ml	eqiupment	20min	**	[16]

*: simple; **: complex; ***: technician; **** skilled technician
